# A randomized, double‐blind, placebo‐controlled trial of camicinal in Parkinson's disease

**DOI:** 10.1002/mds.27259

**Published:** 2017-12-26

**Authors:** Sarah L. Marrinan, Tal Otiker, Lakshmi S. Vasist, Rachel A. Gibson, Bhopinder K. Sarai, Matthew E. Barton, Duncan B. Richards, Per M. Hellström, Dag Nyholm, George E. Dukes, David J. Burn

**Affiliations:** ^1^ Royal Infirmary of Edinburgh, NHS Lothian Edinburgh United Kingdom; ^2^ Newcastle University, Institute of Ageing and Health Newcastle upon Tyne United Kingdom; ^3^ GlaxoSmithKline R&D Stevenage United Kingdom; ^4^ GlaxoSmithKline R&D, Research Triangle Park North Carolina United States; ^5^ GlaxoSmithKline Cambridge United Kingdom; ^6^ Department of Medical Sciences Uppsala University Uppsala Sweden; ^7^ Department of Neuroscience, Neurology Uppsala University Uppsala Sweden; ^8^ Newcastle University, Institute of Neurosciences Newcastle upon Tyne United Kingdom

**Keywords:** Parkinson's disease/parkinsonism, Clinical trials Randomized controlled (CONSORT agreement)

## Abstract

**Background**: Delayed gastric emptying may impair l‐dopa absorption, contributing to motor fluctuations. We evaluated the effect of camicinal (GSK962040), a gastroprokinetic, on the absorption of l‐dopa and symptoms of PD.

**Methods**: Phase II, double‐blind, placebo‐controlled trial. Participants were randomized to receive camicinal 50 mg once‐daily (n = 38) or placebo (n = 20) for 7 to 9 days.

**Results:** l‐dopa exposure was similar with coadministration of camicinal compared to placebo. Median time to maximum l‐dopa concentration was reduced, indicating more rapid absorption of l‐dopa. Camicinal resulted in significant reduction in OFF time (–2.31 hours; 95% confidence interval: –3.71, –0.90), significant increase in ON time (+1.88 hours; 95% confidence interval: 0.28, 3.48) per day, and significant decrease in mean total MDS‐UPDRS score (–12.5; 95% confidence interval: –19.67, ‐5.29). Camicinal treatment was generally well tolerated.

**Conclusions**: PD symptom improvement with camicinal occurred in parallel with more rapid absorption of l‐dopa. This study provides evidence of an improvement of the motor response to l‐dopa in people with PD treated with camicinal 50 mg once‐daily compared with placebo, which will require further evaluation. © 2017 The Authors. Movement Disorders published by Wiley Periodicals, Inc. on behalf of International Parkinson and Movement Disorder Society.

Delayed gastric emptying (GE) affects more than 70% of PD patients.^1^, ^2^ Levodopa is rapidly absorbed in the proximal small bowel. If GE is delayed, the absorption of l‐dopa is impaired and may result in delayed ON and motor fluctuations.^3^, ^4^ Gastroprokinetics may enhance GE, l‐dopa absorption, and motor function.^5^, ^6^, ^7^, ^8^ Camicinal is a motilin receptor agonist, which enhances GE in healthy volunteers and patients with diabetic gastroparesis.^9^, ^10^

The primary outcome of this study was to evaluate the effect of camicinal on l‐dopa pharmacokinetics (PK) in patients with PD. We hypothesized that camicinal would improve GE resulting in increased l‐dopa absorption, earlier l‐dopa peak concentrations, increased drug exposure, and better control of motor and nonmotor symptoms.^11^ We also evaluated the safety of camicinal in PD.

## Materials and Methods

This was a multicenter (11), international (UK, Europe, Australia) phase II randomized, double‐blind, placebo‐controlled study. It was conducted in accordance with the Declaration of Helsinki and registered on www.clinicaltrials.gov (NCT01602549). Eligible patients were randomized 2:1 to receive camicinal (50 mg once‐daily) or placebo.

There was a screening period of 1 to 21 days, a 7‐ to 9‐day treatment period, and a 14‐day follow‐up period.

The primary outcome of the study was change in l‐dopa pharmacokinetics. Secondary outcomes included change in GE, motor, and nonmotor symptoms. Camicinal safety and tolerability were also evaluated.

Initially this was a dose‐ranging study where participants were to receive camicinal 25, 50, or 125 mg. However, after an interim review and concerns about the development potential of the 125‐mg dose, the protocol was amended to compare a single strength of camicinal (50 mg) with placebo. One patient received camicinal 125 mg before this amendment and is included in the adverse events (AEs) summary.

Patients (40‐80 years old) diagnosed with idiopathic PD according to the UK Brain Bank Criteria (modified H & Y stage II‐IV) with suboptimal motor control on l‐dopa‐based treatment were eligible. Motor fluctuations were defined as wearing‐off, peak‐dose dyskinesias, and delayed or absent ON periods that impacted functional status or quality of life. Incapacitating peak‐dose or biphasic dyskinesias were an exclusion criterion. Patients were required to have a stable l‐dopa therapy regimen for at least 4 weeks preceding screening, and therapies were not altered during the study period. Patients were suitable for participation if their GE T1/2 at screening was ≥ 70 minutes, as determined by the ^13^C‐Gastric Emptying Breath Test (GEBT; Cairn Diagnostics, Brentwood, TN). Significant gastric pathology or surgery or use of medications influencing upper gastrointestinal motility within a week of study participation were exclusion criteria.

Patients attended 5 visits: screening, first dosing (day 1), mid‐point review (day 4 ± 1), final dosing (day 8 ± 1), and follow‐up (day 14 ± 2 after final dose).

At the screening, first, and final dosing visits, patients attended in the morning, fasted and in a functionally defined OFF state. Patients took their usual l‐dopa‐based medication (time 0) after the study medication (time ‐90 minutes). They received a standardized GEBT test meal (time ‐10 minutes) and did not ingest any additional food for the 240‐minute study period.

Plasma samples were obtained at predetermined time points for l‐dopa pharmacokinetics analysis; area under the concentration‐time curve (AUC), maximal concentration (Cmax), and time to Cmax (Tmax) were calculated.

The full MDS‐UPDRS was assessed at baseline and days 1 and 8, just before l‐dopa dosing. The MDS‐UPDRS Part III was assessed by blinded raters at 120, 180, and 240 minutes after dosing. Subjects completed a daily motor symptoms diary. AEs were assessed using a symptom diary.

### Statistical Analysis

Analyses were performed on the “All Subjects” population: participants who received at least one dose of study medication.

The planned sample size was calculated using data from Hauser and colleagues.^13^ Assuming the mean (standard deviation) of dose‐normalized l‐dopa AUC_0‐4_ to be 48.65 (18.93) ng.h/mL/mg, corresponding to a coefficient of variation of 38.9%, the planned sample size of 45 subjects (camicinal 30, placebo 15) was powered (90% power, 5% alpha) to detect a 1.5‐fold change in mean AUC_0‐4_.

An interim analysis was performed after 16 completed subjects to assess futility and re‐estimate sample size. At the interim analysis, the probability that the camicinal to placebo fold change in l‐dopa AUC_0‐4_ was calculated. If this probability was greater than 70%, the study would stop. Futility criteria were not met. The sample size was increased to 58. Additional assessments were included in a revised protocol (“ON”/”OFF” symptoms at the screening visit to allow for baseline comparisons and MDS‐UPDRS 3 assessments for evaluation of motor symptoms at the subject defined “ON” time).

#### Pharmacokinetic Analysis

Pharmacokinetic parameters were calculated by noncompartmental methods using Phoenix WinNonlin (v. 6.2; Certara) for baseline and days 1 and 8. l‐dopa exposure parameters (AUC_0‐t_, Cmax) were dose‐normalized by dividing the parameter value by the equivalent l‐dopa dose in mg based on each subject's individual l‐dopa‐based regimen. These parameters were log‐transformed and analyzed using a mixed‐model analysis of variance (ANOVA), from which point estimates and 95% confidence intervals (CIs) for the ratio, “camicinal 50 mg: Placebo” were calculated. Change from baseline to day 8 in l‐dopa Tmax was analyzed using a Wilcoxon rank‐sum test.

#### Efficacy Analysis

An estimation approach was used for all efficacy endpoints. The point estimate and corresponding 95% CI for the difference “camicinal ‐ Placebo” were constructed, using the residual error from the model fitted.

Change from baseline GE T1/2 and changes from baseline in MDS‐UPDRS were analyzed by a mixed‐model ANOVA.

For the purpose of statistical analysis, diary categories of ON without dyskinesia and ON with nontroublesome dyskinesia were combined to a single category referred to as “ON.” The change from baseline in awake hours spent ON and OFF was analyzed separately using an analysis of covariance (ANCOVA) model with the baseline measurement included as a covariate.

## Results

Twenty patients were randomized to placebo and 38 to camicinal. One subject from the placebo group withdrew consent. One patient receiving camicinal was withdrawn because of noncompliance. Baseline demographics and disease characteristics of the groups were comparable.

There was no significant difference in l‐dopa AUC (0‐t) or Cmax when camicinal was coadministered on days 1 or 8. However, the Tmax occurred approximately 23 minutes sooner on day 8 with camicinal versus placebo. Median Tmax on day 8 was reduced from 120 minutes (range, 18‐240) at baseline to 97 minutes (18‐240) in the camicinal group compared to placebo, where Tmax increased from 90 (18‐216) to 120 minutes (30‐210; *P* = 0.186).

GE T1/2 was reduced by 5.3 minutes (95% CI: ‐6.9, 17.6) in the camicinal group compared to placebo from baseline to day 8.

Compared to placebo, treatment with camicinal resulted in a significant improvement from baseline of approximately 13 points (95% CI: 5, 20) in the total MDS‐UPDRS scores. MDS‐UPDRS subsection scores improved from baseline to day 8 in the camicinal group versus placebo; part I (4 points; 95% CI: 2, 5), part II (2 points; 95% CI: 0, 5), and part III (5 points, 95% CI: 0, 10). On day 8, at 4 hours postdose, the mean MDS‐UPDRS part III score was 9 points (95% CI: 3, 15) lower for the camicinal group than the placebo group.

Camicinal resulted in a significant increase in the number of hours/day spent in the ON state (+1.88 hours; 95% CI: 0.28, 3.48) and a significant reduction in time spent in the OFF state (–2.31 hours; 95% CI: –3.71, –0.90) compared to placebo (Table 1).

**Table 1:** Change from baseline to end of treatment period in number of hours spent daily in each symptoms state

	Adjusted Mean (95% CI)		
State	Placebo (n = 17)	Camicinal 50 mg (n = 36)	Adjusted Mean Difference: Camicinal 50 mg ‐ placebo (95% CI)
OFF	0.99 (–0.17, 2.14)	−1.32 (−2.11, −0.53)	−2.31 (−3.71, −0.90)
ON	−0.82 (−2.13, 0.50)	1.06 (0.15, 1.96)	1.88 (0.28, 3.48)
ON without dyskinesia	−0.37 (−2.12, 1.37)	0.73 (−0.47, 1.93	1.11 (−1.01, 3.22)
ON with nontroublesome dyskinesia	−0.47 (−1.52, 0.59)	0.34 (−0.39, 1.06)	0.80 (−0.48, 2.09)
ON with troublesome dyskinesia	−0.12 (−0.38, 0.15)	0.20 (0.02, 0.39)	0.32 (−0.01, 0.64)

The adjusted means and differences were estimated using an ANCOVA model fitting treatment and baseline amount of hours spent daily in ON/OFF state as main effects.

**Figure 1:**
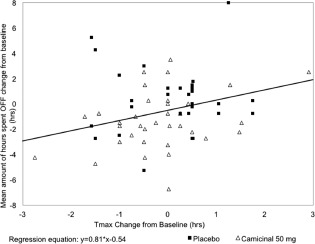
Correlation between change from baseline in OFF time and levodopa Tmax on day 8.

There was a correlation between reduction in l‐dopa Tmax and improvements in OFF time (r = 0.31; *P* < 0.05; Fig. 1). Regression of change from baseline in Tmax against change in OFF time estimates that, on average, a 1‐hour reduction in Tmax led to a 50‐minute reduction in OFF time. A similar correlation was observed between a reduction in Tmax and an increase in ON time.

Overall, camicinal pharmacokinetics were similar to those observed in previously in healthy volunteers and diabetic subjects with gastroparesis.^9^, ^10^

Reported AEs were similar in both groups, the most frequent being headache and fatigue. Two subjects receiving camicinal reported nonfatal serious adverse events: pruritus and circulatory collapse (not related to camicinal).

## Discussion

Although there was similar l‐dopa drug exposure between the two groups, the median l‐dopa Tmax in the camicinal group occurred, on average, 60 minutes earlier than in the placebo group. This suggests that although camicinal co administration did not increase overall l‐dopa exposure, it did result in more rapid time to maximal plasma concentration. However, the precision of the Tmax and Cmax estimates may be low given that the l‐dopa plasma concentrations were assessed only every 30 minutes. This could be further explored in the future with more frequent PK sampling.

Patients who received camicinal reported less OFF and more ON time compared to those receiving placebo, a statistically significant improvement which may also be clinically significant.^14^ Reduction in OFF time was greater than might have been predicted from the modest change in GE, possibly reflecting the heterogeneity of gastric motility, disease progression, or potentially another pharmacological mechanism.

Significant improvements in MDS‐UPDRS scores were observed in patients who received camicinal, most likely explained by the decrease in l‐dopa Tmax.

Tolerability of camicinal 50 mg once‐daily for 7 to 9 days was similar to placebo. Two AEs of dyskinesia occurred in the camicinal group, an effect that could be expected to occur from a more rapid drug absorption (shorter time to Tmax).

Although we found a trend toward more rapid GE in the camicinal group, this was not statistically significant. GE assessed by GEBT over a 4‐hour period may not be of sufficient sensitivity to distinguish subtle changes in emptying rates.

Limitations of this study include inability to precisely assess Tmax or to accurately determine “time to ON” attributed to sampling frequency and study design. Thus, the mechanism(s) by which camicinal leads to motor improvement remains uncertain. With the relatively small sample size and short duration of treatment, it was not possible to determine whether the observed benefits were a true drug effect.

This is the first report that the motilin receptor agonist camicinal may improve the motor response to l‐dopa in PD with motor fluctuations. Camicinal may improve motor fluctuations by shortening the time to peak l‐dopa plasma concentration. Camicinal represents a promising adjunctive therapeutic option worthy of further evaluation in a larger study.

## Author Roles

(1) Research Project: A. Conception, B. Organization, C. Execution; (2) Statistical Analysis: A. Design, B. Execution, C. Review and Critique; (3) Manuscript Preparation: A. Writing of the First Draft, B. Review and Critique.

S.L.M.: 1B, 1C, 2C, 3A, 3B

T.O.: 2A, 2B, 2C, 3B

L.S.V.: 1A, 1B, 1C, 2A, 2B, 2C, 3B

R.A.G.: 1A, 1B, 2C, 3B

B.K.S.: 1B, 1C, 3B

M.E.B.: 1A, 1B, 1C, 3B

D.B.R.: 1B, 1C, 2B, 3B

P.M.H.: 1B, 1C, 3B

D.N.: 1C, 2C,3B

G.E.D.: 1A, 1B, 2A, 2C, 3B

D.J.B.: 1B, 3A, 3B

## Financial Disclosures

T.O. has been an employee of GlaxoSmithKline. L.S.V. has been an employee of GlaxoSmithKline. R.A.G. has been an employee of GlaxoSmithKline. D.B.R. has been an employee of GlaxoSmithKline. P.M.H. has received institutional support from Uppsala University. D.N. has received royalties from Liber AB; lecture fees from AbbVie NordicInfu Care; has received research support from AbbVie, Ipsen, Selanders Foundation, Swedish Knowledge Foundation, Swedish Parkinson's Disease Foundation, and VINNOVA Sweden's innovation agency; is a co‐founder and stock owner in Jemardator AB; and received institutional support from Uppsala University Hospital. D.J.B. has received speaker fees from Profile Pharma; grant support from NIHR, MRC, and Parkinson's UK; and educational material royalties from Henry Stewart Publications and Oxford University Press. SLM has received speaker fees from Parkinson's Academy.
